# Mouse preimplantation embryo responses to culture medium osmolarity include increased expression of CCM2 and p38 MAPK activation

**DOI:** 10.1186/1471-213X-7-2

**Published:** 2007-01-10

**Authors:** Barry Fong, Patricia H Watson, Andrew J Watson

**Affiliations:** 1 Department of Physiology and Pharmacology, The University of Western Ontario London, Ontario, Canada; 2 Department of Physiology and Pharmacology, and Medicine, The University of Western Ontario and Lawson Health Research Institute, London, Ontario, Canada; 3 Department of Physiology and Pharmacology, and Obstetrics and Gynaecology, The University of Western Ontario and Children's Health Research Institute – Lawson Health Research Institute, London, Ontario, Canada

## Abstract

**Background:**

Mechanisms that confer an ability to respond positively to environmental osmolarity are fundamental to ensuring embryo survival during the preimplantation period. Activation of p38 mitogen-activated protein kinase (MAPK) occurs following exposure to hyperosmotic treatment. Recently, a novel scaffolding protein called Osmosensing Scaffold for MEKK3 (OSM) was linked to p38 MAPK activation in response to sorbitol-induced hypertonicity. The human ortholog of OSM is cerebral cavernous malformation 2 (CCM2). The present study was conducted to investigate whether CCM2 is expressed during mouse preimplantation development and to determine whether this scaffolding protein is associated with p38 MAPK activation following exposure of preimplantation embryos to hyperosmotic environments.

**Results:**

Our results indicate that *Ccm2 *along with upstream p38 MAPK pathway constituents (*Map3k3*, *Map2k3*, *Map2k6*, *and Map2k4*) are expressed throughout mouse preimplantation development. CCM2, MAP3K3 and the phosphorylated forms of MAP2K3/MAP2K6 and MAP2K4 were also detected throughout preimplantation development. Embryo culture in hyperosmotic media increased p38 MAPK activity in conjunction with elevated CCM2 levels.

**Conclusion:**

These results define the expression of upstream activators of p38 MAPK during preimplantation development and indicate that embryo responses to hyperosmotic environments include elevation of CCM2 and activation of p38 MAPK.

## Background

Culture medium osmolarity is one of the primary parameters that must be considered when formulating an optimized medium for the production of preimplantation embryos. Even brief exposure of preimplantation embryos to 300 mOsm/kg culture media (in the absence of osmolytes) results in impaired development [1-3]. Most mammalian embryo culture media formulations have employed osmolarities around 250 mOsm/kg. Greater culture medium osmolarities may be employed but only in the presence of osmolytes such as glycine, betaine, proline and glutamine [2,4]. Preimplantation embryos express a number of transporters that serve to regulate and maintain embryonic cell volume [5-7]. In somatic cell systems, activation of p38 MAPK is a common response of these osmoregulatory pathways [8,9].

MAPKs function by propagating extracellular signals via a series of phosphorylation events through sequentially arranged protein kinases, resulting in cellular responses ranging from transcriptional to post-translational events [10]. p38 MAPK consists of four mammalian isoforms including MAPK14/p38α [11], MAPK11/p38β [12], MAPK12/p38γ [13], and MAPK13/p38δ [14]. p38 MAPKs are activated by a variety of environmental stresses and proinflammatory cytokines resulting in phosphorylation of the Thr-Gly-Tyr phosphoacceptor sequence [10,15]. Directly upstream of p38 MAPK, there are at least three dual specificity Thr-Tyr kinases identified that phosphorylate and activate p38: MAP2K3 (or MKK3) [16], MAP2K6 (or MKK6) [17], and MAP2K4 (or MKK4) [16]. Downstream substrates of the p38 MAPKs include protein kinases such as MAPK-activated protein kinase 2 (MAPKAPK2) [18] and p38 regulated/activated kinase (PRAK/MAPKAPK5) [19], as well as several transcription factors including MEF2, CHOP, and ATF2 [10].

The discovery of a class of compounds called cytokine-suppressive anti-inflammatory drugs (CSAIDs) has allowed for the specific pharmacological inhibition of MAPK14/p38α and MAPK11/p38β isoforms [20]. The most extensively characterized CSAIDs are the pyridinyl imidazoles SB203580 [21] and the more potent SB220025 [22]. We have reported that all four p38 MAPK isoforms are expressed throughout mouse preimplantation development [23]. In addition, embryos treated with CSAIDs experience a reversible blockade of development at the 8–16 cell stage which is accompanied by a reversible loss of filamentous actin (F-actin) [23,24]. These results point towards an essential role for MAPK14/11 in directing development of the mouse embryo past the 8–16 cell stage [23-25].

**Figure 1:**
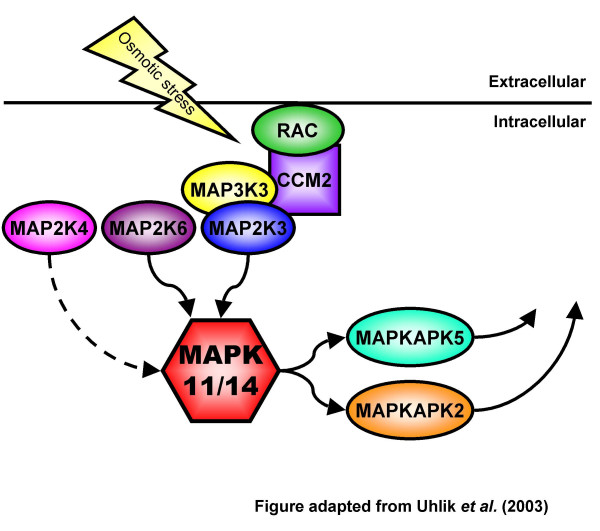
**CCM2 as a Scaffold for p38 MAPK Activation**. As described in [26] the Rho-GTPase, RAC, is recruited to actin membrane ruffles by CCM2 following hyperosmotic stress, facilitating the activation of p38 MAPK. CCM2 acts as a scaffold binding to RAC, MAP3K3, and MAP2K3 to organize these components into a functional signaling module. We have included in this model the two additional upstream MAP2Ks to p38 MAPK: MAP2K6, a specific activator of p38 MAPK related to MAP2K3; and MAP2K4, which primarily activates the JNK/SAPK pathway, but can also phosphorylate p38 MAPK *in vitro*. These two kinases may contribute to activating p38 MAPK in response to hyperosmotic stress but unlikely via interactions with CCM2 [26]. Downstream substrates of p38 MAPK include MAPKAPK2 and MAPKAPK5. Figure adapted from Uhlik et al. [26].

Among its possible roles in the early embryo, p38 MAPK signaling is likely to mediate embryonic responses to hyperosmotic stimuli. Recently, a novel scaffolding protein called **O**smosensing **S**caffold for **M**EKK3 (OSM) was characterized [26]. OSM binds to F-actin, the GTPase, RAC, and the upstream kinases MAP3K3/MEKK3 and MAP2K3 in the p38 MAPK phospho-relay module, recruiting these proteins to sites of active membrane ruffling and newly polymerized actin (Figure 1) [26]. Down-regulation of OSM by RNA interference demonstrated that MAP3K3 and OSM were required for p38 MAPK activation in response to sorbitol-induced hypertonicity [26]. The current mouse gene name for OSM is cerebral cavernous malformation 2 homolog (human) (CCM2).

The present study was conducted to investigate whether CCM2 and upstream p38 MAPK pathway constituents are expressed during preimplantation development and to determine whether changes in CCM2 expression are associated with p38 MAPK activation following exposure of preimplantation embryos to hyperosmotic stimuli. Our results indicate that CCM2 is expressed throughout mouse preimplantation development and its protein levels are elevated in response to hypertonic culture conditions and correlate with increased p38 MAPK activity in the early embryo.

## Results

### Detection of mRNA transcripts encoding *Ccm2, Map3k3, Map2k3, Map2k6*, and *Map2k4 *during mouse preimplantation development

**Figure 2:**
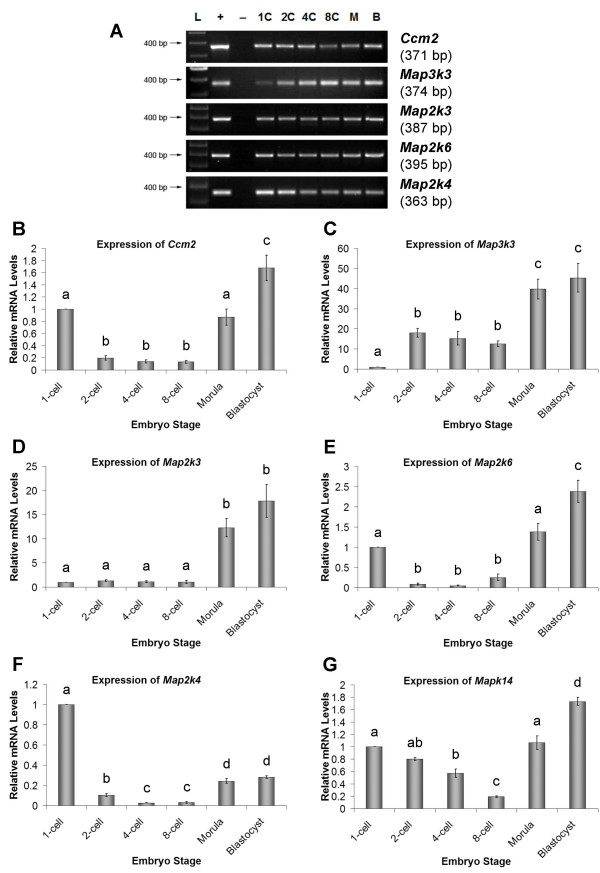
**Detection of mRNA Transcripts encoding p38 MAPK Pathway Constituents and CCM2 during Mouse Preimplantation Development**. RT-PCR products encoding *Ccm2*, *Map3k3*, *Map2k3*, *Map2k6*, and *Map2k4 *were detected in cDNA from one (1) embryo equivalent at timed stages of development 1C, 1-cell; 2C, 2-cell; 4C, 4-cell; 8C, 8-cell; M, morula; B, blastocyst; L, ladder **(A)**. Kidney tissue RT positive controls (+) and no cDNA template negative controls (-) are shown. Representative image of three independent replicates. Quantitative analysis of *Ccm2 ***(B)**, *Map3k3 ***(C)**, *Map2k3 ***(D)**, *Map2k6*, **(E)**, *Map2k4 ***(F) **and *Mapk14 ***(G)**. mRNA transcript levels in a developmental series of mouse preimplantation embryos by Real-Time RT-PCR. Data are normalized to *Luciferase *control (0.025 pg/embryo) and relative to 1-cell target gene mRNA levels. Relative mRNA levels are presented as the mean ± s.e.m. representative of three independent replicates. Bars with different letters represent significant differences in relative mRNA levels between embryo stages (*P *≤ 0.05).

Qualitative RT-PCR methods resulted in the detection of mRNA transcripts encoding *Ccm2*, *Map3k3*, *Map2k3*, *Map2k6 *and *Map2k4 *producing the expected size amplicons of 371 bp, 374 bp, 387 bp, 395 bp and 363 bp respectively throughout mouse preimplantation development in all 3 experimental replicates (Figure 2A). The identity of each RT-PCR product was confirmed through direct sequencing and BLAST^® ^analysis. RT-PCR products amplified using *Ccm2*, *Map3k3*, and *Map2k3 *primers possessed 100% sequence identity with their respective GenBank^® ^mouse nucleotide sequences. RT-PCR products amplified using *Map2k6 *and *Map2k4 *primers possessed 98% and 99% sequence identity with their corresponding GenBank^® ^mouse nucleotide sequences, respectively.

### Relative abundance of mRNAs encoding *Ccm2, Map3k3, Map2k3, Map2k6, Map2k4 *and *Mapk14 *during mouse preimplantation development

*Ccm2 *(Figure 2B) and *Map2k6 *(Figure 2E) mRNA steady state levels were significantly highest at the blastocyst stage (*P *≤ 0.05) and did not vary significantly between the morula and 1-cell stages (*P *> 0.05). However, both *Ccm2 *and *Map2k6 *mRNA levels decreased significantly from 1-cell levels to those observed at the 2-cell, 4-cell, and 8-cell stage (*P *≤ 0.05). *Map3k3 *(Figure 2C) and *Map2k3 *(Figure 2D) steady state mRNA levels were significantly highest at the morula and blastocyst stages (*P *≤ 0.05). In contrast, *Map2k4 *(Figure 2F) steady state mRNA levels were highest at the 1-cell stage and declined significantly at the 2-cell stage (*P *≤ 0.05). *Map2k4 *mRNA levels then remained low throughout preimplantation development, but did increase significantly (*P *≤ 0.05) at the morula and blastocyst stages (Figure 2F). Finally, *Mapk14 *(Figure 2G) steady state mRNA levels were significantly highest (*P *≤ 0.05) at the blastocyst stage. *Mapk14 *mRNA levels gradually declined from the 1-cell stage to reach their lowest level at the 8-cell stage before increasing significantly (*P *≤ 0.05) again at the morula and blastocyst stages.

### Cellular distribution of CCM2, MAP3K3, phosphorylated MAP2K3/MAP2K6 and phosphorylated MAP2K4 proteins during mouse preimplantation development

**Figure 3:**
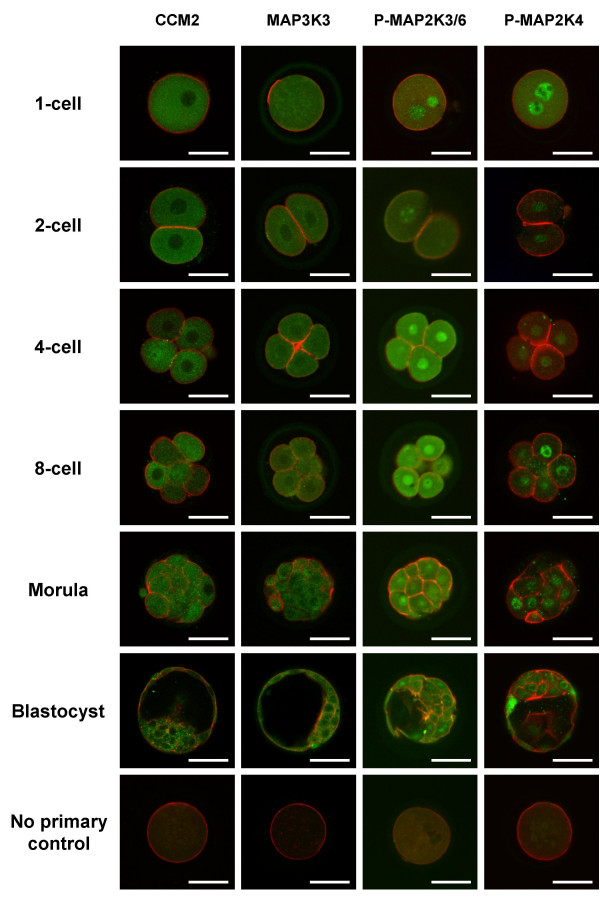
**Immunofluorescence Detection of CCM2, MAP3K3, Phosphorylated MAP2K3/6 and Phosphorylated MAP2K4 during Mouse Preimplantation Development**. Whole-mount indirect immunofluorescence of 1-cell, 2-cell, 4-cell, 8-cell, morula, and blastocyst stage mouse embryos with antisera raised against CCM2 **(CCM2)**, MAP3K3 **(MAP3K3)**, phosphorylated MAP2K3/MAP2K6 **(P-MAP2K3/6) **and phosphorylated MAP2K4 **(P-MAP2K4)**. CCM2 and MAP3K3 immunofluorescence was distributed diffusely throughout the cytoplasm of each blastomere in all embryo stages. Phosphorylated MAP2K3/MAP2K6 (phosphorylated at Ser189/107) immunofluorescence was detected in the cytoplasm and nucleus of each blastomere in each embryo stage. Phosphorylated MAP2K4 (phosphorylated at Thr261) immunofluorescence was primarily localized to the nucleus of each blastomere in each embryo stage. In all confocal micrographs, green indicates positive staining for specific protein and red indicates F-actin (rhodamine-phalloidin). Scale bars represent 50 μm.

CCM2 immunofluorescence was detected in all stages of early mouse development, from the 1-cell to the blastocyst stage (Figure 3 CCM2 1-cell to blastocyst). CCM2 was confined to the cytoplasm of each blastomere, where immunofluorescence was detected diffusely throughout. Similarly, MAP3K3 immunofluorescence was detected in all preimplantation developmental stages (Figure 3 MAP3K3 1-cell to blastocyst). The diffuse distribution of MAP3K3 immunofluorescence throughout the cytoplasm of each blastomere mirrored the distribution of CCM2. Phosphorylated MAP2K3/MAP2K6 (Figure 3 p-MAP2K3/6 1-cell to blastocyst) and phosphorylated MAP2K4 (Figure 3 p-MAP2K4 1-cell to blastocyst) were also detected throughout preimplantation development. Phosphorylated MAP2K3/MAP2K6 was detected in the cytoplasm and the nucleus at all preimplantation stages of mouse development although the nuclear fluorescence was more intense than the cytoplasmic fluorescence. Phosphorylated MAP2K4 was primarily confined to the nucleus of each blastomere throughout preimplantation development (Figure 3 p-MAP2K4 1-cell to blastocyst). In some cases faint phosphorylated-MAP2K4 immunofluorescence was detected in the cytoplasm. This was not consistent and was not restricted to a particular embryo stage (Figure 3 p-MAP2K4 1-cell to blastocyst). For all proteins these distribution patterns were consistently observed in both trophectoderm and inner cell mass cell types of the blastocyst.

### Effect of culture in 1800 mOsm hyperosmotic medium on blastocyst morphology and p38 MAPK activation

**Figure 4:**
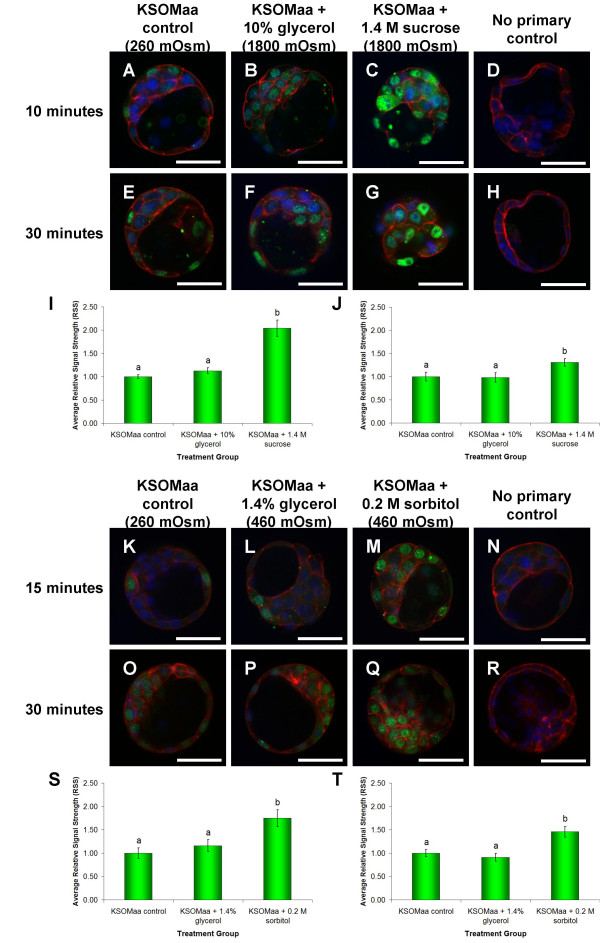
**Immunofluorescence Detection and Quantitation of Phosphorylated MAPKAPK2 Following Culture in 1800 mOsm Medium for 10 Minutes and 30 Minutes**. Phosphorylated MAPKAPK2 was detected in the cytoplasm and the nucleus of all embryos observed following 10 minutes and 30 minutes of treatment. There was a noticeable increase in the level of phosphorylated MAPKAPK2 protein detected in blastocysts cultured in KSOMaa + 1.4 M sucrose **(C, G) **when compared with blastocysts cultured in KSOMaa + 10% glycerol **(B, F) **or KSOMaa only **(A, E) **at both 10 minutes and 30 minutes of treatment. No primary controls are shown (**D, H**). All confocal micrographs are representative images from pools of blastocysts in each treatment group. Green indicates positive staining for MAPKAPK2 proteins (when phosphorylated at Thr334), red indicates F-actin (rhodamine-phalloidin) and blue indicates nuclei (DAPI). Scale bars represent 50 μm. Scion Image analysis applied to FITC immunofluorescence images representing detectable phosphorylated MAPKAPK2 at 10 minutes **(I) **and 30 minutes of treatment **(J)**. Relative signal strengths are presented as the mean ± s.e.m. representative of three independent replicates. Bars with different letters represent significant differences in relative signal strength between treatment groups (*P *≤ 0.05). **Immunofluorescence Detection and Quantitation of Phosphorylated MAPKAPK2 Following Culture in 460 mOsm Medium for 15 Minutes and 30 Minutes**. Phosphorylated MAPKAPK2 was detected in the cytoplasm and the nucleus of all embryos observed following 15 minutes and 30 minutes of treatment. There was a noticeable increase in the level of phosphorylated MAPKAPK2 protein detected in blastocysts cultured in KSOMaa + 0.2 M sorbitol **(M, Q) **when compared with blastocysts cultured in KSOMaa + 1.4% glycerol **(L, P) **or KSOMaa only **(K, O) **at both 15 minutes and 30 minutes of treatment. No primary controls are shown **(N, R)**. All confocal micrographs are representative images from pools of blastocysts in each treatment group. Green indicates positive staining for MAPKAPK2 proteins (when phosphorylated at Thr334), red indicates F-actin (rhodamine-phalloidin) and blue indicates nuclei (DAPI). Scale bars represent 50 μm. Scion Image analysis resulted in the quantification of FITC immunofluorescence representing detectable phosphorylated MAPKAPK2 polypeptides at 15 minutes **(S) **and 30 minutes of treatment **(T)**. Relative signal strengths are presented as the mean ± s.e.m. representative of three independent replicates. Bars with different letters represent significant differences in relative signal strength between treatment groups (*P *≤ 0.05).

We initially employed treatment with high culture osmolarity (i.e. 1800 mOsm) to explore the effects of an extreme treatment paradigm on phospho-MAPKAP2 fluorescence. Blastocyst culture in KSOMaa media containing 10% glycerol or 1.4 M sucrose resulted in an instantaneous decrease in blastocyst volume. Those placed in medium + 10% glycerol displayed a rapid recovery (5 minutes) back to normal volume, however, this recovery was not observed in embryos placed in 1.4 M sucrose medium (data not shown). Phospho-MAPKAPK2 immunofluorescence increased in blastocysts cultured in KSOMaa + 1.4 M sucrose for 10 minutes (Figure 4C) or for 30 minutes (Figure 4G) when compared to blastocysts cultured in KSOMaa + 10% glycerol for 10 minutes (Figure 4B) or 30 minutes (Figure 4F), or when compared to blastocysts cultured in normal KSOMaa control media (control) for 10 minutes (Figure 4A) or 30 minutes (Figure 4E). Quantitation of phospho-MAPKAPK2 immunofluorescence by Scion Image analysis demonstrated that 10 minutes of culture in KSOMaa + 1.4 M sucrose resulted in a significant increase (*P *≤ 0.05) in relative signal strength (RSS; mean ± s.e.m.) when compared to normal KSOMaa cultured controls and also blastocysts cultured in KSOM + 10% glycerol [2.04 ± 0.17 (n = 14), 1.00 + 0.04 (n = 33), and 1.13 ± 0.06 (n = 25), respectively] (Figure 4I). The RSS of blastocysts cultured in KSOMaa + 10% glycerol displayed was not significantly different from normal KSOMaa cultured controls (Figure 4I). The same outcomes between treatment groups were observed at 30 minutes (Figures 4J). Blastocysts cultured in KSOMaa + 1.4 M sucrose displayed a significant increase (*P *≤ 0.05) in RSS levels compared to levels obtained for the normal KSOMaa cultured controls and blastocysts cultured in KSOMaa + 10% glycerol [1.31 ± 0.08 (n = 20), 1.00 ± 0.09 (n = 19), and 0.982 ± 0.103 (n = 18), respectively] (Figure 4J).

### Effect of 460 mOsm hyperosmotic treatment on blastocyst morphology and p38 MAPK activation

To investigate the effects of reduced levels of hyperosmolarity on phospho-MAPKAPK2 fluorescence we next conducted the following experiment using culture medium adjusted to 460 mOsm. Exposure of cultured blastocysts to a 1.4% glycerol or 0.2 M sorbitol medium did not visibly affect blastocyst volume (data not shown). While no perceptible differences in blastocyst volume were observed between embryos following 15 minutes and 30 minutes of 460 mOsm hyperosmotic treatment, variations in the levels of phospho-MAPKAPK2 fluorescence were detected (Figure 4K–R). Following 15 minutes of exposure to 460 mOsm hyperosmotic medium treatments, blastocysts cultured in KSOMaa + 0.2 M sorbitol displayed a significant increase (*P *≤ 0.05) in RSS of phosphorylated MAPKAPK2 immunofluorescence (1.75 ± 0.18, n = 33) when compared to blastocysts cultured in normal KSOMaa culture medium (1.00 ± 0.11, n = 33) or KSOMaa + 1.4% glycerol (1.16 ± 0.13, n = 34) (Figure 4S). The RSS of phosphorylated MAPKAPK2 immunofluorescence in blastocysts cultured in KSOMaa + 1.4% glycerol did not differ from KSOMaa only controls (Figure 4S). At the 30 minute time-point, the RSS of blastocysts cultured in KSOMaa + 0.2 M sorbitol was significantly higher (*P *≤ 0.05) than that observed for blastocysts cultured in KSOMaa + 1.4% glycerol and KSOMaa only controls [1.46 ± 0.12 (n = 35), 0.911 ± 0.082 (n = 35), and 1.00 ± 0.08 (n = 33), respectively] (Figure 4T). Once again there was no significant difference in the RSS displayed by blastocysts cultured in the glycerol medium and normal KSOMaa medium controls (Figure 4T).

### Effect of 460 mOsm hyperosmotic treatment on *Ccm2 t*ranscripts and CCM2 protein

**Figure 5:**
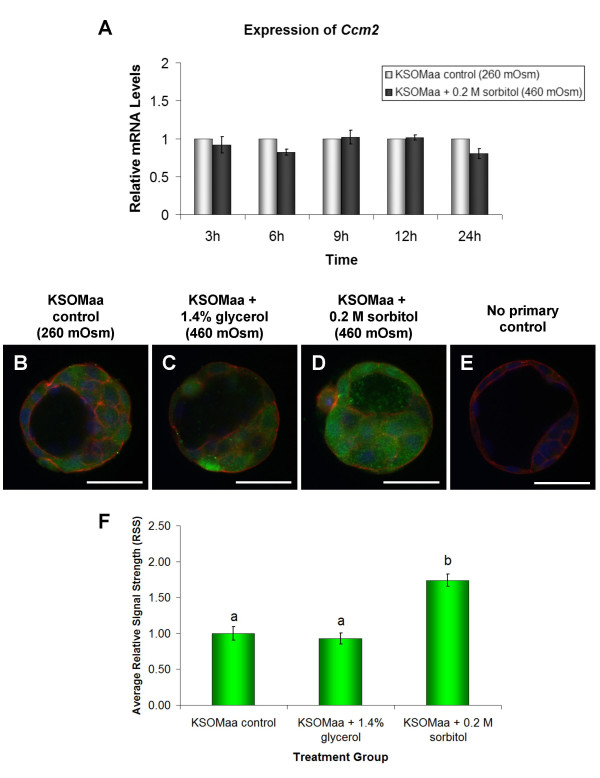
**Effect of 460 mOsm Hyperosmotic Treatment on *Ccm2 *mRNA and CCM2 Immunofluorescence**. Quantitative analysis of *Ccm2 *in blastocyst stage mouse embryos following treatment in KSOMaa + 0.2 M sorbitol (approximately 460 mOsm) and KSOMaa only (approximately 260 mOsm) **(A)**. Data is normalized to *Luciferase *and relative to mRNA transcript levels detected in the KSOMaa only control group at each individual time-point of treatment. Relative mRNA levels are presented as the mean ± s.e.m. representative of three independent replicates. No significant differences in relative mRNA transcript levels were observed (*P *≤ 0.05). There was a noticeable increase in the level of CCM2 immunofluorescence detected in blastocysts cultured for 15 minutes in KSOMaa + 0.2 M sorbitol **(D) **when compared with blastocysts cultured in KSOMaa + 1.4% glycerol **(C) **or KSOMaa only **(B)**. No primary control group is shown (**E**). Green indicates positive staining for CCM2 proteins (when phosphorylated at Thr334), red indicates F-actin (rhodamine-phalloidin) and blue indicates nuclei (DAPI). Scale bars represent 50 μm. Scion Image analysis resulted in the quantification of FITC immunofluorescence representing detectable CCM2 polypeptides **(F)**. Relative signal strengths are presented as the mean ± s.e.m. Bars with different letters represent significant differences in relative signal strength between treatment groups (P ≤ 0.05).

To explore a possible mechanism for the induction of p38 MAPK following incubation in hyperosmotic medium we next examined the influence of exposure to hyperosmotic medium on *Ccm2 *expression. We employed the 460 mOsm culture medium treatment for these experiments since we had now established that this treatment was sufficient to significantly increase phospho-MAPKAPK2 immunofluorescence levels. Cultured blastocyst stage mouse embryos were exposed to hyperosmotic treatment for 3, 6, 9, 12, or 24 hours in KSOMaa + 0.2 M sorbitol (460 mOsm) and compared with cultured blastocysts in normal KSOM medium (control group, 260 mOsm). No significant differences (*P *> 0.05) in relative *Ccm2 *mRNA transcript levels were observed between blastocysts cultured in the KSOMaa + 0.2 M sorbitol and blastocysts cultured in the normal KSOMaa medium control group at any of the investigated time-points (Figure 5A). Despite this outcome, we next investigated whether CCM2 protein levels may be affected. Whole-mount immunofluorescence methods employing anti-CCM2 antiserum were applied to mouse blastocysts cultured in KSOMaa + 1.4% glycerol (460 mOsm), KSOMaa + 0.2 M sorbitol (460 mOsm), and normal KSOMaa medium (260 mOsm) for 15 minutes (Figure 5B–E). Results from the quantification of CCM2 immunofluorescence are presented as relative signal strength (RSS) (mean ± s.e.m.) (Figure 5F). Following 15 minutes of treatment in 460 mOsm medium, blastocysts cultured in KSOMaa + 0.2 M sorbitol (n = 26) displayed a significant increase in RSS of CCM2 immunofluorescence when compared to blastocysts cultured in normal KSOMaa medium (n = 26) (1.74 ± 0.09 vs. 1.00 ± 0.10, respectively) or KSOMaa containing 1.4% glycerol (n = 27) (1.74 ± 0.09 vs. 0.926 ± 0.077, respectively) (Figure 5F) (*P *≤ 0.05). Blastocysts cultured in KSOMaa + 1.4% glycerol did not display a significant difference in RSS from blastocysts cultured in normal KSOMaa medium (Figure 5F).

## Discussion

Our overall objective was to increase our understanding of the intracellular signaling pathways that respond to the external environment and regulate preimplantation development. Our recent characterization of the expression of all four isoforms of p38 MAPK (MAPK14/p38α, MAPK11/p38β, MAPK12/p38γ, and MAPK13/p38δ) and a number of its downstream substrates provided the foundation for our current studies [23]. We have recently demonstrated a requirement for p38 MAPK signaling during preimplantation development by characterizing the reversible developmental blockade that occurs at the 8- to 16-cell stage following inhibition of MAPK14/p38α and MAPK11/p38β isoforms [23]. Furthermore, p38 MAPK regulates F-actin polymerization during preimplantation development via phosphorylation of downstream substrates MAPKAPK2 and PRAK/MAPKAPK5, and subsequently through the small heat shock proteins, HSPB1/2 [23,24]. Our present study has extended these initial investigations into p38 MAPK function during preimplantation development by addressing the upstream events in the three-tiered MAPK pathway module. Our first objective was to characterize the expression of regulatory kinase gene products upstream of p38 MAPK during preimplantation development. In this regard we have demonstrated that transcripts and proteins encoding all known MAPKKs upstream of p38 MAPK (i.e. MAP2K3, MAP2K6, and MAP2K4) are present throughout mouse preimplantation development. Moreover, we have also identified transcripts and proteins encoding the MAP3K, MAP3K3, as well as the novel scaffolding protein, CCM2.

Each gene of interest displayed a consistent increase in transcript abundance as embryo development advanced towards the blastocyst stage. There was a significant increase in relative mRNA levels from the 8-cell stage to the morula and blastocyst stages for all transcripts investigated. These results suggest that mRNAs for p38 MAPK pathway constituents are rapidly accumulating in the post 8-cell stage embryo and this observation correlates well with outcomes from p38 MAPK inhibition studies which suggest that p38 MAPK signaling is required to maintain development beyond the 8–16 cell stage [23-25]. Of particular note is the nearly 40-fold increase over 1-cell levels in *Map3k3 *expression at the morula stage, and the 12-fold increase over the same period in *Map2k3 *expression which indicates that MAP3K3 and MAP2K3 may be the primary upstream activators of p38 MAPK during preimplantation development. Conversely, the relatively low expression of *Map2k4 *mRNA transcripts past the 1-cell stage suggests a reduced role for this kinase during early development. Our results indicate that all of these gene products are derived from both oogenetic (maternal) and embryonic origins since they are found in both pre- and post-maternal zygotic transition (MZT) stage embryos. The predominant pattern observed for transcripts encoding p38 MAPK upstream regulators was one where mRNA levels clearly increased with advancing cleavage stage. This accumulation of mRNAs with advancing embryo stage could only occur via contributions from embryonic transcriptional activity.

At the protein level, the CCM2 and MAP3K3 cytoplasmic distribution pattern observed following application of whole-mount indirect immunofluorescence was as expected considering the role of CCM2 as an actin binding scaffold protein and MAP3K3 as an upstream MAP3K that is responsive to extracellular stimuli at the cell surface. The diffuse cytoplasmic distribution mirrors the localization pattern displayed by these proteins in HEK293 and COS7 cell lines [26,27]. We also determined the distribution of phosphorylated MAP2K3/MAP2K6 proteins and MAP2K4 proteins throughout mouse preimplantation development. The use of antisera recognizing the phosphorylated forms of these proteins allowed us to not only characterize their distribution throughout preimplantation development but also report their activation during preimplantation development. Activated MAP2Ks were detected in the cytoplasm and nucleus of embryos collected from the 1-cell to the blastocyst stage, indicating that the p38 MAPK pathway is activated from the earliest stages of development onward. While phospho-MAP2K3/MAP2K6 proteins were detected in the cytoplasm and the nucleus, phospho-MAP2K4 proteins were confined to the nuclei, suggesting that p38 MAPK phosphorylation by MAP2K4 may occur predominantly in the nucleus. This nuclear localization is supported by studies that investigated the activation of JNK/SAPK in P19 embryonic carcinoma cells [28]. MAP2K4 was detected in both the nucleus and cytoplasm of differentiated P19 cells, but only in the nucleus of undifferentiated P19 cells [28]. Other studies have established that JNK/SAPK and MAP2K4 are activated and translocated into the nucleus in response to cellular stress [29,30]. In total, these results establish the presence of all components of the Rac-CCM2-MAP3K3-MAP2K3 signaling complex throughout preimplantation development when coupled with our previous studies that determined Rac1 expression during mouse preimplantation development (Figure 1) [31].

Since it is well known that the roles played by p38 MAPK extend well beyond the regulation of the actin cytoskeleton our present study investigated whether embryonic p38 MAPK signaling is affected by exposure to hyperosmotic stimuli as is reported for somatic cells. Our results clearly demonstrate that hyperosmotic treatment during culture increased p38 MAPK activity as assessed by increased phosphorylation of its specific downstream substrate, MAPKAPK2. These findings are supported by numerous studies that have described the responsiveness of this pathway to hyperosmotic stress, and have indicated that increases in p38 MAPK activity are a common cellular response to hyperosmotic stimuli (reviewed by [9]. While the increased p38 MAPK activity and phosphorylation of MAPKAPK2 in itself is not a novel discovery, the responsiveness of p38 MAPK to hyperosmotic stress has not until now been demonstrated to occur during preimplantation development. One of the most exciting outcomes from our study is the differential activation of p38 MAPK that occurred in response to the solute compound used to increase the osmolarity of embryo culture media.

At both 1800 mOsm and 460 mOsm hyperosmotic treatment conditions, the addition of glycerol to KSOMaa culture medium did not result in a significant increase in phospho-MAPKAPK2 immunofluorescence in blastocysts. This is in contrast to the 1.4 M sucrose (1800 mOsm) and 0.2 M sorbitol (460 mOsm) treatments in which a significant increase in phospho-MAPKAPK2 immunofluorescence was detected at two treatment time-points. We propose that this difference in p38 MAPK activation is a result of the alleviation of osmotic stress and restoration of osmotic equilibrium across the cell membrane due to aquaporin (AQP; water channel) mediated glycerol permeability. This likelihood is supported by our previous studies which demonstrated the selective permeability of water and glycerol, but not sucrose, through apical and basolateral AQPs located in the blastocyst trophectoderm [32,33]. Since glycerol can permeate the cell membrane relatively freely via AQPs, the initial osmotic gradient (from 260 mOsm to 1800 mOsm or 460 mOsm) produced by the addition of glycerol to culture media is rapidly reversed in these treatment groups, alleviating "osmotic stress" that might induce p38 MAPK activity. This also permits the observed re-expansion of blastocyst volume that does not occur with 1.4 M sucrose treatment [32]. The osmotic gradient produced by 1.4 M sucrose (1800 mOsm) or 0.2 M sorbitol (460 mOsm) persists for the duration of treatment, enough to induce p38 MAPK activity. Our results may therefore have an impact on studies directed at optimizing embryo cryopreservation protocols as the cyroprotectant used (ie glycerol or other) would be expected to have differential effects on p38 MAPK activation which may influence embryo survival and recovery post-thaw. Investigation of this possibility could result in improved outcomes following embryo cryopreservation.

Interestingly, hyperosmotic treatment of cultured rat astrocytes by mannitol or sorbitol increases the expression of AQP4 and AQP9 mRNAs and proteins, which is suppressed by treatment with the p38 MAPK inhibitor SB203580 [34]. Glycerol treatment, however, had no effect on the expression of these two AQPs [34]. Similar to our study, this was attributed to the absence of an osmotic gradient as a result of glycerol movement across cell membranes. Other AQP family members were also differentially expressed in sucrose, sorbitol, or mannitol treatment (in which an osmotic gradient *is *established) [35,36]. Mouse preimplantation embryos are capable of regulating AQP mRNA abundance in response to environmental changes in osmolarity and changes to blastocoel volume following puncture [37], however, the potential role of p38 MAPK activity in mediating these events was not investigated. Based upon our results, we would hypothesize that a prolonged shift in osmotic gradient induces p38 MAPK activity, which in turn regulates AQP expression as a compensatory mechanism in preimplantation embryos. This would also represent an important direction for future studies to pursue.

By quantifying relative signal strength of phospho-MAPKAPK2 immunofluorescence, we observed a 2-fold increase in p38 MAPK activity following 1.4 M sucrose treatment for 10 minutes and a 1.75-fold increase following 0.2 M sorbitol treatment for 15 minutes. A 2-fold increase in p38 MAPK activity is consistent with results observed in other studies [38-40]. These studies describe rapid (within seconds to minutes) activation of p38 MAPK in response to hyperosmotic treatment through the addition of various solutes to culture conditions [38-40]. This activation typically reaches peak levels following 15 minutes of treatment, but elevated p38 MAPK activity is sustained for at least 60 minutes [38,40]. It should be noted that while the conditions of hyperosmotic treatment and the methods of assessing p38 MAPK activity vary between these studies, the results are remarkably consistent. Tilly et al., [39]described a 2-fold increase in the enzymatic activity of MAPKAPK2 at 10 minutes after 820 mOsm mannitol-induced hyperosmotic treatment in human intestine 407 cells [39]; while Watts et al. demonstrated in rat medullary thick ascending limb (MTAL) kidney cells a 2.3-fold increase in ATF-2 (downstream transcription factor of p38 MAPK) phosphorylation with 0.3 M mannitol following 15 minutes treatment [40]. Furthermore, p38 MAPK activity peaked at 15 minutes of 0.2 M sucrose treatment in Chinese hamster ovary cells (CHO-K1) with a 2-fold increase in p38 MAPK phosphorylation [38]. Our findings are consistent with these studies since, following peak p38 MAPK activation, there is a steady decline in activity over time. At both experimental osmolarities employed in our study we detected a decrease in RSS (of sucrose/sorbitol groups) from the first time-point (10 minutes and 15 minutes, for 1800 mOsm and 460 mOsm respectively) to the second time-point (30 minutes, for both). Other studies have described peak p38 MAPK activity at approximately 15 minutes in response to hyperosmotic stress. This would suggest that the first time-point in our treatments is near peak levels of p38 MAPK activity, with some of that activity decreased by 30 minutes in the preimplantation mouse embryo. The marginal difference in RSS between 1.4 M sucrose (at 1800 mOsm) and 0.2 M sorbitol (at 460 mOsm) is somewhat surprising. One would expect that an 1800 mOsm osmotic gradient would activate p38 MAPK to a much greater extent than a 460 mOsm gradient. A possible explanation could be that despite the difference between 1800 mOsm and 460 mOsm that we may perceive, the embryo may consider both stimuli as equivalent when translating them into responses for activating p38 MAPK. In this regard it is also possible that 460 mOsm represents a near-maximal stimulus of p38 MAPK activation and thus levels beyond this would not result in any further increase in p38 MAPK activity.

Though changes were not detected in localization or steady state mRNA transcript levels of CCM2 following hyperosmotic treatment, our results did reveal a significant increase in CCM2 immunofluorescence in blastocysts treated for 15 minutes in 0.2 M sorbitol (460 mOsm), but not 1.4% glycerol (460 mOsm). These results suggest a solute specificity in the induction of CCM2, which agrees with the induction of p38 MAPK activity and phosphorylation of MAPKAPK2 described above. Uhlik et al. [26] demonstrated the specificity of OSM/CCM2 to hyperosmolar stimuli by comparing sorbitol and anisomycin treatment, however alternative means of varying treatment osmolarity (i.e. using cell permeable solutes such as glycerol) were not attempted in this study. Interestingly, the 1.74-fold increase in CCM2 immunofluorescence we observed mirrored the 1.75-fold increase in p38 MAPK activity assessed by MAPKAPK2 phosphorylation. Uhlik et al. reported a 2.01-fold increase in MEKK3 kinase activity in HEK293 cells treated with 0.2 M sorbitol for 15 minutes [26]. The kinetics of hyperosmolarity-induced MAP3K3 activation reported in that study closely mirrored that of endogenous p38 MAPK activation in the HEK293 cells. An increase in detectable CCN2 protein without an increase in mRNA levels suggests that 0.2 M sorbitol treatment increases CCM2 expression at the translational level, requiring de novo protein synthesis. An alternative explanation for this increase in detectable CCM2 protein may be in the regulation of scaffold protein stability. If 0.2 M sorbitol treatment results in the increased binding of CCM2 protein with its scaffolding partners, it could result in decreased proteolysis and degradation of CCM2 protein. This would explain the significantly elevated RSS of CCM2 immunofluorescence detected in the sorbitol-treated blastocysts when compared with glycerol-treated and control blastocysts. Either of these possibilities would require further study, as very little is currently known about the translational regulation of CCM2 protein, nor is it clear how CCM2-p38 MAPK interactions may be regulated in general. While our study has presented a novel discovery in the induction of CCM2 in response to hyperosmotic treatment of preimplantation embryos, much work remains to fully characterize CCM2 activity and regulation during this period of development.

## Conclusion

In conclusion, we have demonstrated for the first time that transcripts and polypeptides encoding MAP3K3, MAP2K3, MAP2K6, MAP2K4 and CCM2 are expressed and localized throughout mouse preimplantation development. We have discovered that p38 MAPK activity is regulated by exposure to hyperosmotic stimuli, and that the response to hyperosmotic stress in the early embryo includes increased CCM2 levels. These outcomes provide a basis for understanding the mechanisms controlling osmotic induction of p38 MAPK activity during preimplantation development. The outcomes therefore further our knowledge of the regulation of intracellular signaling pathways within the mammalian embryo and understanding of how culture environments can affect embryo development. The preimplantation embryo is highly sensitive to the environment in which it develops. With the extension of embryo culture to the blastocyst stage emerging as a routine treatment paradigm in human infertility clinics, it becomes even more important that we study the short-term and long-term effects of culture on embryo development. Our results may therefore contribute to advancements in the development of improved embryo culture systems with the ultimate goal of increasing our ability to produce healthy embryos for embryo transfer.

## Methods

### Superovulation and mouse embryo collection

Female CD-1 (6–8 weeks of age, Charles River Laboratories, Saint-Constant, QC, Canada) or MF-1 mice (4–5 weeks of age, Harlan Sprague Dawley, Indianapolis, IN, USA) were superovulated by intraperitoneal (IP) injection of 10 IU pregnant mare serum gonadotropin (PMSG; Intervet Canada Ltd., Whitby, ON, Canada) followed 48 hours later by 10 IU human chorionic gonadotropin (hCG; Intervet Canada Ltd., Whitby, ON, Canada) prior to mating with CD-1 males (Charles River Laboratories, Canada) as described [23,24,41]. Embryos were collected at specified times following hCG injection, which correspond to appropriate cleavage stages: 1-cell zygotes 24 hr post-hCG; 2-cell, 48 hr; 4-cell, 60 hr; 8-cell, 65–68 hr; morulae, 80–85 hr and blastocyst, 90 hr. Embryos were collected by either flushing reproductive tracts with flushing medium 1 (1-cell – 8-cell stages) or flushing medium II (morulae and blastocysts) [42]. Embryos were washed several times in flushing medium and collected in pools to be either: 1) frozen and stored at -80°C for RNA extraction; 2) fixed for immunofluorescence analysis of protein distribution; or 3) cultured in 20 μL drops of EmbryoMax^® ^KSOMaa (potassium simplex optimized medium with amino acids) Liquid Mouse Embryo Media (Chemicon International – Specialty Media, Temecula, CA, USA) [43] under mineral oil and maintained in culture under 5% CO_2 _in air atmosphere at 37°C. Animal care and treatment followed protocols established by the UWO Animal Care Committee.

### RNA extraction

Total RNA was extracted from pools of 20 or 40 preimplantation stage mouse embryos or positive control tissue (mouse kidney). Samples were frozen and stored at -80°C in 500 μl of TRI Reagent^® ^(Molecular Research Center Inc., Cincinnati, OH, USA) with 5 μL of Polyacryl Carrier (Molecular Research Center Inc., Cincinnati, OH, USA) following collection. In samples used for quantitative Real-Time reverse transcription and polymerase chain reaction (RT-PCR) analysis, 0.025 pg/embryo of exogenous Luciferase Control RNA (Promega Corporation, Madison, WI, USA) containing a 30-base poly(A) tail was added to each pool of embryos prior to RNA extraction.

### Reverse Transcription and Polymerase Chain Reaction (RT-PCR)

Embryo total RNA was reverse transcribed (RT) using Random Primers (Invitrogen Life Technologies, Burlington, ON, Canada) and RNaseOUT™ Ribonuclease Inhibitor (Invitrogen Life Technologies, Burlington, ON, Canada) along with Sensiscript RT (Qiagen Inc., Mississauga, ON, Canada) according to the manufacturer's suggested protocol. Following one hour incubation, the sample was diluted to a concentration of 1 embryo equivalent per μL (embryo/μL) and subjected to PCR amplification of H2A histone family, member Z (*H2afz*), *Luciferase*, or both to determine the efficiency of the RNA extraction and reverse transcription prior to investigation of expression of the target genes through standard and Real-Time PCR amplification. Polymerase chain reaction (PCR) was performed using one embryo equivalent of cDNA from each stage per reaction in a 50 μL volume consisting of 1.0 U of Platinum^® ^*Taq *DNA Polymerase (Invitrogen Life Technologies, Burlington, ON, Canada), 1X PCR Reaction Buffer (Invitrogen Life Technologies, Burlington, ON, Canada), 1.5 mM MgCl_2_, 0.2 mM dNTPs, and 1.0 μM of each PCR primer. Primer pairs were designed and synthesized (Sigma-Aldrich Canada Ltd., Oakville, ON, Canada) for *Ccm2*, *Map3k3*, *Map2k3*, *Map2k6*, *Map2k4*, and *H2afz *based on mouse nucleotide sequences in the Ensembl mouse transcript database. Primers for *Luciferase *were designed and synthesized (Sigma-Aldrich Canada Ltd., Oakville, ON, Canada) based on the nucleotide sequence used to generate the Luciferase Control RNA (Promega Corporation, Madison, WI, USA), as obtained from the manufacturer. All gene-specific primer pairs are listed in Table 1. Qualitative PCR amplification reactions were performed using a Techne Touchgene Gradient DNA thermal cycler (Techne Inc., Burlington, NJ, USA). The reaction was initiated at 95°C for 5 minutes, followed by 40 cycles consisting of denaturation at 95°C for 50 seconds, re-annealing primers to target sequence at 60°C for 50 seconds, and primer extensions at 72°C for 50 seconds. Each amplification reaction was terminated with a 10 minute final extension at 72°C. PCR products were resolved on a 2.0% agarose gel containing 1.0 μg/mL ethidium bromide (Invitrogen Life Technologies, Burlington, ON, Canada) in 1X TAE buffer (40 mM Tris, 40 mM acetate, 1 mM EDTA). The identity of all PCR products was confirmed by sequence analysis (DNA Sequencing Facility, Robarts Research Institute, London, ON, Canada). PCR reactions were repeated a minimum of three times using cDNA prepared from embryos at each of the indicated stages and isolated from a minimum of three separate developmental series. Positive (tissue cDNA: heart, liver, and kidney) and negative control (no cDNA template) samples were included for each primer set in every experiment.

**Table 1:** PCR Primer Sequences for Amplification of *Ccm2*, *Map3k3*, *Map2k3*, *Map2k6*, *Map2k4*, *H2afz*, and *Luciferase*

**Gene product**	**Primer**	**Primer Sequence**	**Size (bp)**	**Ensembl Transcript ID**
*Ccm2*	5'	TCTGCTCAGTCTGTCTGCCTA	371	ENSMUST00000000388
	3'	CAAATATTGCTCGGTCCAGAA		
*Map3k3*	5'	CCATCCTTCAGGAAATCACAA	374	ENSMUST00000002044
	3'	CAATTGATGGGAGCACTAGGA		
*Map2k3*	5'	AGCCTATGGGGTGGTAGAGAA	387	ENSMUST00000019076
	3'	CCTTCCTTGTTGATGAGGACA		
*Map2k6*	5'	AGATGACCTGGAGCCGATAGT	395	ENSMUST00000019076
	3'	GCTTGACGTCTCGATGGATAA		
*Map2k4*	5'	TGGAGCTCATGTCTACCTCGT	363	ENSMUST00000046963
	3'	CGTACAATGTGATCCCCAAAC		
*H2afz*	5'	CTCACCGCAGAGGTACTTGAG	375	ENSMUST00000041045
	3'	ATGCAGAAATTTGGTTGGTTG		
*Luciferase*	5'	TTGACAAGGATGGATGGCTAC	354	N/A
	3'	GTTTTCCGGTAAGACCTTTGC		

### Custom TaqMan^® ^gene expression assay design for real-time PCR

The Custom TaqMan^® ^primer and probe sets for *Ccm2 *and *Luciferase *were designed using the Assays-by-Design File Builder program (Applied Biosystems, Foster City, CA, USA). The probe sequence for CCM2 was designed against a target site 847 bp into the full-length sequence obtained from the Ensembl mouse transcript database (Transcript ID: ENSMUST00000000388). The probe sequence for luciferase was directed against a target site 550 bp into the full-length sequence used to generate the Luciferase Control RNA (Promega Corporation, Madison, WI, USA). The target site specifies an approximate location for generation of a TaqMan^® ^probe, and each target site was verified to be unique by performing BLAST^® ^analysis. Dual-labeled probes were synthesized (Applied Biosystems, Foster City, CA, USA) to contain the reporter dye 6-carboxyfluorescein (6-FAM) at the 5' end and a non-fluorescent quencher dye at the 3' end.

### Quantitative real-time PCR analysis

Real-Time PCR reactions were performed using the ABI PRISM^® ^7900HT sequence detection system (Applied Biosystems, Foster City, CA, USA) and TaqMan^® ^Gene Expression Assays (Applied Biosystems, Foster City, CA, USA). Pre-designed and pre-optimized commercially available TaqMan^® ^Gene Expression Assays for MEKK3/Map3k3 (Assay ID: Mm00803725_m1), MKK3/Map2k3 (Assay ID: Mm00435950_m1), MKK6/Map2k6 (Assay ID: Mm00803694_m1), MKK4/Map2k4 (Assay ID: Mm00436508_m1), p38α/Mapk14 (Assay ID: Mm00442497_m1), were used along with the Custom TaqMan^® ^Gene Expression Assays described above for *Ccm2 *and *Luciferase*. The PCR reaction mixture (50 μL) contained 25 μL of TaqMan^® ^Universal PCR Master Mix (2X concentration, containing AmpliTaq Gold^® ^DNA Polymerase, AmpErase^® ^UNG, dNTPs with dUTP, Passive Reference 1, and optimized buffer components; Applied Biosystems, Foster City, CA, USA), 2.5 μL of the appropriate 20X TaqMan^® ^Gene Expression Assay (see above), 10 μL of embryo cDNA (at a diluted concentration of 0.1 embryo/μL) corresponding to one embryo equivalent per reaction, and 12.5 μL of HyPure™ Molecular Biology Grade Water (HyClone, Logan, UT, USA). Thermal cycling conditions were 50°C for 2 minutes and 95°C for 5 minutes, followed by up to 60 cycles of 95°C for 15 seconds, and a combined annealing extension stage, 60°C for 1 minute. Each reaction was performed in triplicate on ABI PRISM^® ^96-Well Optical Reaction Plates (Applied Biosystems, Foster City, CA, USA). For each gene of interest, a minimum of three sets of embryo developmental series were analyzed.

Relative quantification of target gene expression levels was performed using the comparative C_T _(threshold cycle) method (ABI PRISM^® ^Sequence Detection System, version 2.1, Applied Biosystems, Foster City, CA, USA). The quantification was normalized to the control luciferase RNA levels [44]. Within the log linear phase region of the amplification curve, the difference between each cycle was equivalent to a doubling of the amplified product of the PCR. The ΔC_T _value was determined by subtracting the control C_T _value for each sample from the target gene C_T _value of the sample. Calculation of ΔΔC_T _used either the 1-cell or control sample as a standard. Fold-changes in the relative mRNA expression of the target gene were determined using the formula 2^-ΔΔCT^.

### Antisera

Rabbit antisera raised against the phosphorylated forms of phospho-MKK3/MKK6 (Ser189/207), phospho-SEK1/MKK4 (Thr261), and phospho-MAPKAPK2 (Thr334) were used (Cell Signaling Technologies Inc., Danvers, MA, USA). The anti-MEKK3 antiserum used was a mouse monoclonal antibody (BD Biosciences Pharmingen, San Diego, CA, USA). The rabbit polyclonal anti-OSM/CCM2 antiserum was a gift generously donated by Dr. Gary L. Johnson (University of North Carolina at Chapel Hill) [26]. All primary antisera were used at a dilution of 1:100, with the exception of anti-OSM/CCM2 at a dilution of 1:200, from the commercial or provided stock concentration. Primary antibodies were labeled using fluorescein (FITC)-conjugated donkey anti-rabbit secondary antisera (Jackson ImmunoResearch Laboratories Inc., West Grove, PA, USA) and FITC-conjugated donkey anti-mouse secondary antisera (Jackson ImmunoResearch Laboratories Inc., West Grove, PA, USA).

### Whole-mount indirect immunofluorescence and confocal microscopy

Mouse preimplantation stage embryos were collected and processed for application of whole-mount immunofluorescence as described [23,24,32,41]. Embryo pools were fixed in 2% paraformaldehyde in PBS, washed in PBS and then either processed immediately for whole-mount indirect immunofluorescence or stored at 4°C in Embryo Storage Buffer (1X PBS + 0.9% sodium azide) for up to 3 weeks. Fixed embryos were permeabilized and blocked concurrently by room temperature incubation in Embryo Blocking Buffer (0.01% Triton X-100 + 5% Normal Donkey Serum in 1X PBS) for 1 hour followed by one wash in fresh 1X PBS for 30 minutes at 37°C. Embryos were incubated with primary antisera at a 1:100 or 1:200 dilution in Antibody Dilution/Wash Buffer (ADB: 0.005% Triton X-100 + 1% Normal Donkey Serum in 1X PBS) at 4°C overnight. Embryos were then washed 3 times for 20–30 minutes in ADB at 37°C and incubated with FITC-conjugated secondary antibodies (Jackson ImmunoResearch Laboratories Inc., West Grove, PA, USA) at 1:200 dilution in ADB overnight at 4°C. To visualize F-actin localization and to stain nuclear DNA, the first 30 minute wash in ADB following secondary antibody incubation included rhodamine-conjugated phalloidin (Sigma-Aldrich Canada Ltd., Oakville, ON, Canada), diluted to 1:200 from 5 μg/mL stock solution, and DAPI (Sigma-Aldrich Canada Ltd., Oakville, ON, Canada), diluted to 1:2000 from 1 mg/mL stock solution. Fully processed embryos were mounted onto glass slides in a drop of FluoroGuard™ Anti-Fade Reagent (BioRad Laboratories Canada Ltd., Mississauga, ON, Canada). Immunofluorescence imaging employed a Zeiss Axiovert 100 inverted microscope equipped with a Zeiss LSM 410 laser-scanning module and computer system equipped with Zeiss LSM software (Carl Zeiss Inc., Thornwood, NY, USA).

### Image analysis and quantification of immunofluorescence intensity

To quantify immunofluorescence results we employed the method developed and reported by [45]. All microscope and image capture settings remained constant during the digital capture of confocal micrographs between embryos and between treatment groups. Acquired micrographs were saved in TIFF image format and processed using Adobe Photoshop CS2 (Adobe Systems Inc., San Jose, CA, USA), for recognition, selection and separation of the desired chromogen signal. Quantitative analysis began by separation of the green image channel representing FITC fluorescence of phospho-MAPKAPK2 from the red (representing rhodamine-phalloidin-labelled F-actin) and blue (representing DAPI-stained nuclei) channels. The green channel image was converted to "Grayscale", discarding the other two color channels to ensure the signal remaining represented only FITC fluorescence. The overall luminance of each pixel in the converted image corresponded to the intensity of FITC fluorescence. Each converted micrograph was then inverted so that gray and black pixels represented areas of FITC immunofluorescence on a white background and saved as a new TIFF image file for Scion Image analysis.

The Scion Image program, Version 4.0.3.2 (Scion Corporation, Frederick, MD, USA), is a freely distributed commercial software program that mimics the performance of NIH Image program for quantification of the chromogen signal strength [45]. The grayscale inverted micrographs were opened in Scion Image and following the methods of [45], the mean density of the chromogen signal strength (SS) of each image was measured and recorded. The average SS from at least three micrographs representing no primary antibody controls was subtracted from the SS values of the measured images from each treatment group to produce an adjusted relative SS value. Relative signal strength (RSS) values were obtained by a ratio comparing the adjusted SS values of each treatment to the average adjusted SS of the control group.

### Hyperosmotic treatment of cultured blastocyst stage mouse embryos

Eight cell stage mouse embryos were flushed from the oviducts of timed-pregnant female mice, washed, pooled in groups of 20, and cultured in 20 μL drops of KSOMaa until the blastocyst stage (approximately 90–95 hours post-hCG for cultured embryos) prior to hyperosmotic treatment. Culture medium osmolarity was adjusted by the addition of glycerol, sucrose, or sorbitol to KSOMaa embryo culture. Treatment groups and their respective osmolarities were: (*i*) KSOMaa only (control, approximately 260 mOsm), (*ii*) KSOMaa + 10% glycerol (Sigma-Aldrich Canada Ltd., Oakville, ON, Canada) (approximately 1800 mOsm), (*iii*) KSOMaa + 1.4 M sucrose (VWR BDH Chemicals, Mississauga, ON, Canada) (approximately 1800 mOsm), (*iv*) KSOMaa + 1.4% glycerol (approximately 460 mOsm), and (*v*) KSOMaa + 0.2 M sorbitol (Sigma-Aldrich Canada Ltd., Oakville, ON, Canada) (approximately 460 mOsm). The osmolarity of the embryo culture medium was tested by freezing-point depression using the Advanced^® ^Model 3320 Micro-Osmometer (Advanced Instruments Inc., Norwood, MA, USA). For Real-Time PCR analysis, embryos were treated for 3, 6, 9, 12, or 24 hours prior to determination of transcript levels by real-time RT-PCR as described above. For whole-mount indirect immunofluorescence assays, embryos were treated for times ranging from 10 to 30 minutes and then were processed for whole-immunofluorescence methods as described above.

### Statistical analysis

Statistical analysis was performed using SPSS^®^, Version 14.0 (SPSS Inc., Chicago, IL, USA) or SigmaStat^® ^2.0 (Jandel Scientific Software, San Rafael, CA, USA) software packages. Real-Time PCR results are presented as the mean ± s.e.m. for relative mRNA transcript levels from three independent replicates. Real-time RT-PCR data were square-root transformed, subjected to one-way analysis of variance (ANOVA), and followed by Tukey's Multiple Comparison Test or non-parametric Mann-Whitney Rank Sum Test. Results from Scion Image analysis are presented as the mean ± s.e.m. for relative signal strength (RSS) from three independent replicates with the number of embryos in each treatment group indicated. All data was tested for homogeneity of variances by Levene's Test for Equality of Variances. In instances of equal variance, data was subjected to one-way ANOVA followed by Fisher's Least Significant Different (LSD) test for comparing three means of unequal group size. When equal variances were not observed, data was subjected to Welch's variance-weighted ANOVA followed by the Games-Howell (GH) Post-hoc Test for Multiple Comparisons, appropriate for situations of unequal (or equal) sample sizes and unequal or unknown variances. For all data analysis, *P *≤ 0.05 was considered statistically significant.

## Authors' contributions

BF participated in the design of the study, performed the PCR, immunofluorescence and hyperosmotic response experiments and drafted the manuscript. PHW assisted in conducting the Real-Time RT-PCR analysis, experimental design, analysis of outcomes and provided critical review of the results and manuscript contents. AJW directed the study, provided critical review of the results, co-authored the manuscript and acquired funding for the research program. All authors read and approved the final manuscript.
